# Chemoinformatics in the New Era: From Molecular Dynamics to Systems Dynamics

**DOI:** 10.3390/molecules21030071

**Published:** 2016-03-03

**Authors:** Guanyu Wang

**Affiliations:** Department of Biology, South University of Science and Technology of China, 1088 Xueyuan Rd., Shenzhen 518055, China; wanggy@sustc.edu.cn; Tel.: +86-755-88018215

**Keywords:** chemoinformatics, molecular dynamics simulation, systems dynamics simulation, drug lead optimization

## Abstract

Chemoinformatics, due to its power in gathering information at the molecular level, has a wide array of important applications to biology, including fundamental biochemical studies and drug discovery and optimization. As modern “omics” based profiling and network based modeling and simulation techniques grow in sophistication, chemoinformatics now faces a great opportunity to include systems-level control mechanisms as one of its pillar components to extend and refine its various applications. This viewpoint article, through the example of computer aided targeting of the PI3K/Akt/mTOR pathway, outlines major steps of integrating systems dynamics simulations into molecular dynamics simulations to facilitate a higher level of chemoinformatics that would revolutionize drug lead optimization, personalized therapy, and possibly other applications.

Chemoinformatics is inherently related to dynamics. By using molecular dynamics’ (MD) simulations to solve major problems in medicinal chemistry, together with other computational and informational techniques, chemoinformatics has been very useful in drug discovery and personalized medicine [1]. Data obtained from MD simulations can be further processed by computational techniques such as chemical graph theory, similarity analysis, clustering, data mining, and machine learning, which generate new information and knowledge; and make better decisions faster in the area of drug lead identification and optimization [2,3,4]. These include storage, indexing, and search for information relating to chemical compounds. For storage of chemical information, virtual libraries of compounds (drugs, natural products, diversity-oriented synthetic products) are generated in various ways to explore chemical space and hypothesize novel compounds with desired properties. For example, a library of molecules occupying druglike chemicals was constructed by using chemoinformatic tools to train transition probabilities of a Markov chain on authentic classes of compounds, and then using the Markov chain to generate novel compounds that were similar to the training database [5]. To search chemical compounds efficiently, virtual screening techniques are widely used, which involves computationally screening *in silico* libraries of compounds, by means of various methods such as docking, to identify members likely to possess desired properties such as biological activities against a given target.

In the virtual library, a compound is associated with its structural information and physico-chemical properties, which can be integrated by some model of regression or classification to predict the compound’s biological activity when considered as a drug candidate. Quantitative structure–activity relationship (QSAR) is a widely used such model [6], which relates a set of predictor variables either to the potency of the response variables (regression), or to a categorical value of the response variables (classification). The predictors consist of physico-chemical properties or theoretical molecular descriptors of chemicals. The response variable is usually a biological activity (e.g., the concentration of a substance required to give a certain biological response). QSAR models first summarize a hypothetical relationship between chemical structures and biological activity in a data-set of chemicals, on the basis of which the activities of new chemicals can be predicted. If the response variable is a chemical property, then the models are called QSPR, namely quantitative structure–property relationship [7].

Because the aforementioned simulations are largely limited to the molecular level, it is highly desired that systems dynamics’ (SD) simulations become routine practices of chemoinformatics so as to facilitate applications such as drug lead optimization. Indeed, a biological function cannot be realized by one or few kinds of molecules; it is the collective effect of a myriad of biomolecules. In other words, it is the complex network of interactions between molecules in the normal living cell that ultimately leads to the biocomplexity that we observe: sophisticated normal functions, complex disease progressions, and so on. To understand these complexities, the entire system has to be modeled (with certain simplifications) and simulated to uncover a novel “response variable,” such as the underlying mechanism of the progression of a disease. We shall call it *response object* to distinguish it from the conventional response variable, and more importantly, to stress that it might be more complex and informative than merely a number or a combination of numbers. As more and more such response objects are identified, and/or methods to seek them are standardized, SD simulations will become a common component of chemoinformatics.

This objective is no longer unreachable, in consideration of recent advances in systems biology, in particular dynamical modeling of biomolecular networks. Systems biology often yields deeper understanding of physiologic/pathologic processes and sheds new light on many aspects of therapeutic interventions, such as optimal drug combinations, optimal timing of therapy, better prophylaxis, diagnostics, and prognostics. SD simulations may uncover overarching mechanisms or systems-level properties that emerge from physico-chemical properties of individual molecules, especially when combined with MD simulations. These uncovered mechanisms may serve as response objects, after some mathematical formulation. With the capability of characterizing complex regulatory events that are temporarily transient and spatially inhomogeneous, these response objects may be superior to a handful of response variables formulated by conventional QSAR.

With response objects rendered by SD simulations, an important component of future drug discovery and design will be in terms of efficient tuning of a response object into its normal range of functioning. To rectify the aberrant response objects, nodal molecules in the network should be extensively perturbed to test their effects on the response objects, and SD simulations will be needed for economic and efficient screening of efficacious combinations of drugs. Therefore, instead of indiscriminately targeting many nodes in the network, SD simulations will help us to find combinations of few crucial molecules to which response objects are sensitive. Furthermore, mathematical techniques such as optimal control theory can be applied to accelerate the process [8]. These mathematical techniques are certainly helpful. However, even without them, SD simulations alone can, in principle, be able to achieve our goals. In this way, optimal combinations of drug targets can be identified, which correspond to multivalent drugs with low total dosage. This will greatly reduce toxicity and potential drug resistance, equivalent to achieving synergistic effects among the drug components [9].

The thriving of SD simulations will never put MD simulations in the shadows. In fact, the MD/SD hybrid simulations will become indispensable and important. SD simulation can identify crucial drug targets, but whether or not a drug target is actually druggable needs to be judged by MD simulations. Therefore, SD simulations should not just find one drug candidate, but a set of many drug candidates, which are then screened sequentially by MD simulations to determine those that are druggable.

Using PI3K/Akt/mTOR pathway as our model system, we have made progress towards this endeavor [8,10,11,12,13]. The PI3K/Akt/mTOR pathway (Figure 1) is a major cellular signaling pivot in the cellular response to extracellular stimuli, including insulin, insulin-like growth factor (IGF), epidermal growth factor (EGF) and fibroblast growth factor (FGF). It carries out a large spectrum of cellular functions such as glucose and lipid metabolism, cell growth, proliferation, and survival, cell migration and polarity [13]. Deregulations of the pathway may lead to a variety of complex diseases such as cancer, type 2 diabetes, neurodegenerative diseases, and muscle hypotrophy [11,14]. Therefore, dynamical modeling and simulation of the PI3K/Akt/mTOR pathway can provide great insights into its normal function and various pathologic changes. More importantly, we have formulated an ideal response object whose modes of variation have clear correspondence to molecular regulations. By studying the response object’s modes of variation, some regulatory mechanisms of the pathway have been revealed. The response object is the stimulus-response curve of protein kinase Akt, denoted by $A\left(I\right)$, where *A* is the percentage of the phosphorylated Akt (pAkt), namely $A=\left[pAkt\right]/\left[Akt_{total}\right]$. The symbol *I* can represent different factors: insulin, IGF, or EGF, or any other relevant stimuli. See Figure 2 and Figure 3 for examples of $A\left(I\right)$. It turns out that the stimulus-response curve is either a graded curve (represented by the curve in the blue-hatched region in Figure 3) or a bistable curve (represented by curves in the other two regions in Figure 3). The bistable curve is characterized by the pair ($I_{\text{on}}$, ΔI), where $I_{\text{on}}$ is the switch-on threshold; $\Delta I=I_{\text{on}}-I_{\text{off}}$ is the difference between $I_{\text{on}}$ and the switch-off threshold $I_{\text{off}}$. Note that ΔI has been called the hysteresis width [12]. The blank region in Figure 3 is characterized by $I_{\text{off}}>0$. The red-hatched region in Figure 3 is characterized by $I_{\text{off}}<0$, which causes the “jump-down” part of the stimulus-response curve being cut off by the vertical axis and causes constitutive activation of Akt. This response object has intuitive biological meanings. For example, “the minimal concentration of a substance to elicit a given biological response” is a typical response variable in a QSAR model, but the reason why such a value should exist is usually not explained. In the present example, SD simulation has revealed that “the minimal stimulus concentration to elicit Akt activation” is determined by a threshold ($I_{\text{on}}$) built in the PI3K/Akt/mTOR pathway.

Mathematical analysis of the dynamical model revealed three composite parameters (α, β, *K*) that affect the response object in three orthogonal ways [10,13,14]. The parameter α represents the feedback strength from pAkt to insulin receptor substrate (IRS); it represents the net effect of positive and negative feedbacks (see Figure 1). The parameter β corresponds to PI3K activity. The parameter *K* has the expression

(1) $$K=\frac{K_{m}}{{\left[Akt\right]}_{total}}$$

where ${\left[Akt\right]}_{total}$ is the total concentration of Akt and $K_{m}=\left(k_{off}+k_{cat}\right)/k_{on}$ is the Michaelis constant of the reaction of Akt phosphorylation. In fact, there is another *K* that corresponds to Akt dephosphorylation. For simplicity, the two *K* values are often assumed the same [10]. It turns out that *K* determines overall sensitivity of A(I). The smaller *K* is, the more sensitive A(I). Figure 2A–C show that: as *K* decreases, the stimulus-response curves become more and more sensitive (*i.e.*, more and more like switches). For parameters α and β, we discovered an interesting decoupling under the limit condition K=0: α affects ΔI but does not affect $I_{\text{on}}$ (Figure 2C); β affects $I_{\text{on}}$ but does not affect ΔI (Figure 2D). When K≠0, the decoupling is not exact but is approximately true as long as *K* keeps small. These discoveries, especially the differential and decoupled roles played by α, β, and *K*, would be very useful to determine how different molecule targeting should be combined.

The parameter space spanned by *K* and α gives a more intuitive global picture (Figure 3). Each point in the diagram represents a given set of (α, β, *K*) values (note that β is always fixed throughout the diagram, e.g., β=1), which completely determines the response object $A\left(I\right)$. By using techniques from nonlinear dynamics, two dividing curves (red and blue) are discovered that divide the diagram into three regions [10]. The response objects in the same region are of the same kind, which is qualitatively different from the other two regions. In the upper region, the response objects are all irreversible switches (bistability with $I_{\text{off}}<0$): once the stimulus exceeds $I_{\text{on}}$, Akt becomes fully activated and remains so even after the complete withdrawal of the stimulus. In other words, the deactivation of Akt is out of control of the stimulus signal. In the middle region, the response objects are all reversible bistable switches: Akt activation and deactivation are both controlled by the stimulus signal. In the lower region, the response objects are all graded curves.

**Figure 1:**
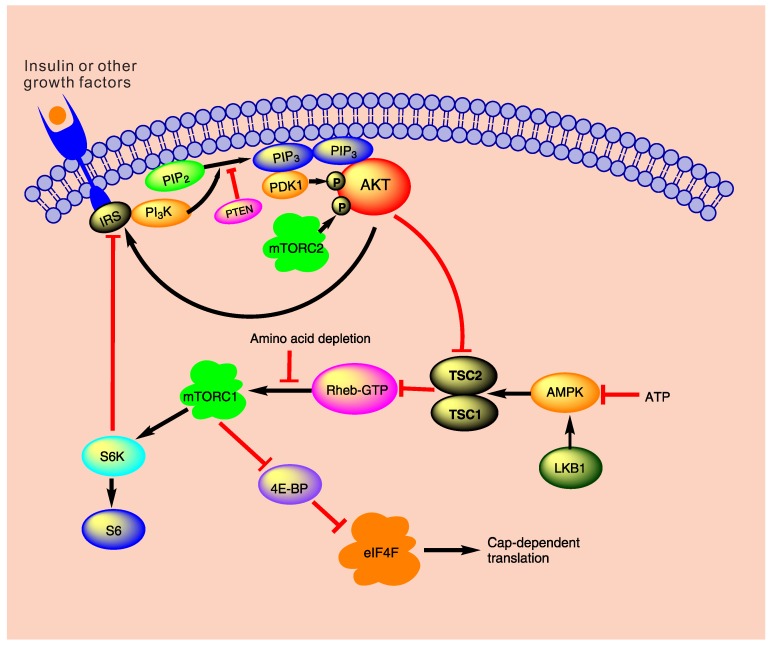
The PI3K/Akt/mTOR pathway. Upon insulin stimulation, the insulin receptor phosphorylates IRS at tyrosine sites, which, in turn, activates PI3K. PI3K then converts PIP2 into PIP3. PIP3 recruits Akt to the membrane, where Akt is phosphorylated by the enzymes PDK1 and mTORC2. Note the two fedback loops: one is the positive feedback from pAkt directly to IRS; the other is the negative feedback from pAkt through mTORC1 to IRS.

The parameter space can be explained in different ways. If the discussion is restricted to the healthy state, then the parameter space may be used to distribute different types of cells; or cells of the same type under different physiologic conditions. For example, different cells may occupy different regions as their normal physiologic states. As environmental conditions change, a cell may transit to another region in order to adapt to the new environment. If the discussion is about changes between various physiologic/pathologic states, the parameter space can be regarded as a division of different such states. For example, a typical normal cell’s (*K*, α) value may be located within the middle region. It has been proposed that bistability possesses many desired properties for normal cell functioning [15]. In particular, it was proved that bistability is the optimal mode of regulation to control homeostasis [8]. With other fine properties (e.g., high sensitivity) taken into account, we propose that the green ellipsoid in Figure 3 indicates parameters that render normal responses. The upper region corresponds to a state of high cancer risk. Indeed, Akt is an oncoprotein (it promotes glucose uptake, cell growth and proliferation, cell survival, and other things that cancer enjoys); and the constitutive activation of Akt would certainly benefit carcinogenesis. The lower region corresponds to relative insensitivity to stimulus. If the stimulus is insulin, then it is a kind of insulin resistance that may underline pathogenesis of type 2 diabetes. That is, if the majority of myocytes’ insulin response is located at the lower region, then the person has a high risk of type 2 diabetes. Finally, it should be noted that Figure 3 is about *K* and α, which does not take account of the change of β. Therefore, even if a cell’s state locates within the green ellipsoid, the cell may still undergo pathologic changes, due to an abnormal β value. Figure 2D has already demonstrated this possibility: the decrease of β causes right-shift of the response object. The enlarged threshold $I_{\text{on}}$ is also a cause of insulin resistance and potential diabetes, although the underlying mechanism is different from the state being in the lower region.

**Figure 2:**
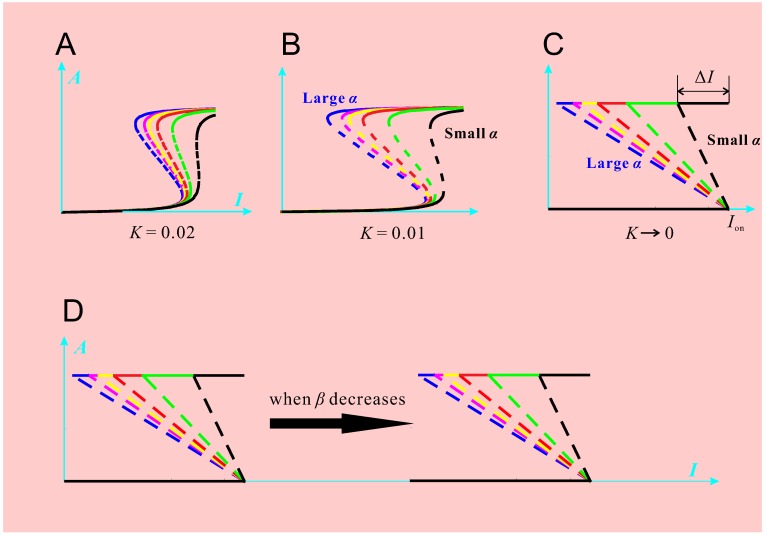
Response objects under various parameter conditions. The parameter α represents the feedback strength from pAkt to IRS. The parameter β corresponds to PI3K activity. The parameter *K* has the expression $K_{m}/\left[Akt_{total}\right]$, where $K_{m}$ is the Michaelis constant of Akt phosphorylation. (**A**) A family of response objects with the same K=0.02 and β=1 but with different α values. The blue (black) curve has large (small) α value; (**B**) The same family of response objects with K=0.01 and β=1; (**C**) The same family of response objects with K=0 and β=1; (**D**) The decrease of β causes right-shift of the response objects.

Our SD simulations have yielded valuable information about drug discovery and optimization for targeting aberrant signaling of the PI3K/Akt/mTOR pathway. The first insight is that one should be cautious when targeting total Akt for cancer therapy. Akt is a prominent oncoprotein and its inhibition has sound rationale [16]. Akt can be inhibited directly (by suppressing gene expression of Akt and thus reducing total Akt) and indirectly (by suppressing Akt phosphorylation). For the direct inhibition, the drug RX-0201 is clinically available [17]. Our SD simulations, however, have shown the importance of total Akt abundance in maintaining a person’s well-being [10]. This is somewhat counterintuitive because it appears that a higher Akt level corresponds to a higher chance of cancer. One would expect that the normal pAkt level should maintain within some median range to tradeoff the risks of cancer and diabetes. Our simulations suggested that what really matters is not about the pAkt level being ‘high or low’, but about how it switches ‘between high and low’. It turns out that a tightly regulated total Akt abundance is critical to sensitivity of the response. Equation (1) tells us that the smallness of *K* depends on the largeness of [Akt]${}_{total}$, namely the total Akt concentration. Therefore, direct Akt inhibition would increase *K* and reduce sensitivity (the direction from Figure 2C to A). Besides sensitivity, other fine properties such as robustness and adaptivity also depend on the abundance of total Akt [10]. Our results thus highlight the indirect inhibition of Akt. Because PI3K is a crucial promoter of Akt phosphorylation, targeting PI3K proved to be an effective approach of indirect Akt inhibition [18]. The second insight is that inhibition of mTOR, a popular drug target, should be accompanied by PI3K inhibition to reduce the side effects [14]. By inhibiting mTOR, growth and proliferation of cancer cells are slowed down. Despite the sound rationale, the method had only modest and unpredictable success in clinical trials [19]. Our simulation results can explain this phenomenon [14]. The parameter α negatively correlates with mTOR activity. Thus mTOR inhibition would increase α and consequently increase ΔI. This would make Akt deactivation more difficult. The prolonged Akt activation would certainly benefit cancer cells. To solve this problem, one can inhibit PI3K to reduce the parameter β, whereby the response object $A\left(I\right)$ shifts to the right (Figure 2D), increasing the chance of Akt deactivation.

**Figure 3:**
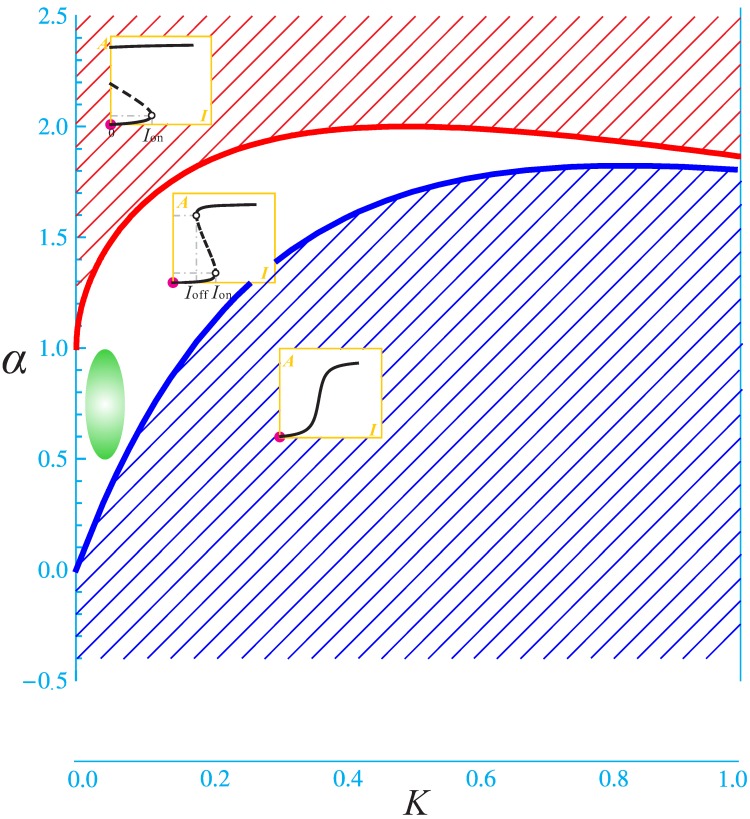
Parameter space of *K* *versus* α with β=1 **fixed.** The space is divided into three regions, which from top down contains irreversible switches, reversible bistable switches,and graded responses.

Although SD simulations hold great promise as novel forefront tools of chemoinformatics to facilitate various applications of chemoinformatics such as drug lead optimization, much more work has to be done so that global information obtained from SD simulations can promote more efficient use of local chemoinformation for drug design and optimization. As the power of today’s “omics” technologies allow for more detailed molecular description, as SD simulation techniques allow for more detailed characterizations of systems-level control mechanisms, as MD/SD hybridization grows in sophistication, and as computers become more powerful, chemoinformatics will have an exciting, hitherto unimagined future: chemical compounds will no longer be profiled simply in terms of physico-chemical properties but also in terms of their impacts on systems-level properties such as sensitivity, robustness, and tunability. Combinations of compounds will also be profiled to give more detailed local information, and for the sake of global integration.

## References

[1] Xu J., Hagler A. (2002). Chemoinformatics and drug discovery. Molecules.

[2] Brown F. (2005). Editorial opinion: Chemoinformatics-a ten year update. Curr. Opin. Drug Discov. Dev..

[3] Willett P. (2013). Fusing similarity rankings in ligand-based virtual screening. Comput. Struct. Biotechnol. J..

[4] Franco P., Porta N., Holliday J.D., Willett P. (2014). The use of 2D fingerprint methods to support the assessment of structural similarity in orphan drug legislation. J. Cheminf..

[5] Kutchukian P.S., Lou D., Shakhnovich E.I. (2009). FOG: Fragment Optimized Growth algorithm for the de novo generation of molecules occupying druglike chemical space. J. Chem. Inf. Model..

[6] Roy K., Narayan Das R. (2014). A review on principles, theory and practices of 2D-QSAR. Curr. Drug Metab..

[7] Tenorio-Borroto E., Ramirez F.R., Speck-Planche A., Cordeiro N.D., Luan F., Gonzalez-Diaz H. (2014). QSPR and Flow Cytometry Analysis (QSPR-FCA): Review and New Findings on Parallel Study of Multiple Interactions of Chemical Compounds with Immune Cellular and Molecular Targets. Curr. Drug Metab..

[8] Wang G. (2012). Optimal homeostasis necessitates bistable control. J. R. Soc. Interface.

[9] Araujo R.P., Liotta L.A., Petricoin E.F. (2007). Proteins, drug targets and the mechanisms they control: The simple truth about complex networks. Nat. Rev. Drug Discov..

[10] Wang G. (2010). Singularity analysis of the AKT signaling pathway reveals connections between cancer and metabolic diseases. Phys. Biol..

[11] Wang G., Krueger G.R. (2010). Computational analysis of mTOR signaling pathway: Bifurcation, carcinogenesis, and drug discovery. Anticancer Res..

[12] Wang G. (2014). Raison d’être of insulin resistance: The adjustable threshold hypothesis. J. R. Soc. Interface.

[13] Li T., Wang G. (2014). Computer-aided targeting of the PI3K/Akt/mTOR pathway: Toxicity reduction and therapeutic opportunities. Int. J. Mol. Sci..

[14] Wang G. (2013). Analysis of Complex Diseases: A Mathematical Perspective.

[15] Tyson J.J., Albert R., Goldbeter A., Ruoff P., Sible J. (2008). Biological switches and clocks. J. R. Soc. Interface.

[16] Simioni C., Martelli A.M., Cani A., Cetin-Atalay R., McCubrey J.A., Capitani S., Neri L.M. (2013). The AKT inhibitor MK-2206 is cytotoxic in hepatocarcinoma cells displaying hyperphosphorylated AKT-1 and synergizes with conventional chemotherapy. Oncotarget.

[17] Pal S.K., Reckamp K., Yu H., Figlin R.A. (2010). Akt inhibitors in clinical development for the treatment of cancer. Expert Opin. Investig. Drugs.

[18] Sabbah D.A., Simms N.A., Brattain M.G., Vennerstrom J.L., Zhong H. (2012). Biological evaluation and docking studies of recently identified inhibitors of phosphoinositide-3-kinases. Bioorg. Med. Chem. Lett..

[19] Guertin D.A., Sabatini D.M. (2007). Defining the role of mTOR in cancer. Cancer Cell.

